# Public health contributions of entomological surveillance of West Nile virus (WNV) and other mosquito-borne arboviruses in a context of climate change

**DOI:** 10.14745/ccdr.v50i09a02

**Published:** 2024-09-05

**Authors:** Bouchra Bakhiyi, Alejandra Irace-Cima, Antoinette Ludwig, Miarisoa Rindra Rakotoarinia, Christian Therrien, Isabelle Dusfour, Ariane Adam-Poupart

**Affiliations:** 1 Department of Biological Risks, Institut national de santé publique du Québec (INSPQ), Montréal, QC; 2 School of Public Health of the Université de Montréal (ESPUM), Université de Montréal, Montréal, QC; 3 Public Health Risk Sciences Division, National Microbiology Laboratory, Public Health Agency of Canada, Saint-Hyacinthe, QC; 4 Research Group on Epidemiology of Zoonoses and Public Health, Faculty of Veterinary Medicine, Université de Montréal, Saint-Hyacinthe, QC; 5 Laboratoire de santé publique du Québec, Institut national de santé publique du Québec, Sainte-Anne-de- Bellevue, QC; 6 Independent medical entomologist, Montpellier, France

**Keywords:** mosquitoes, surveillance, arbovirus, public health, scoping review

## Abstract

**Background:**

Climate change is likely to increase the risk of human transmission of arboviruses endemic to Canada, including West Nile virus (WNV), Eastern equine encephalitis virus (EEEV) and California serogroup virus (CSV), calling for enhanced surveillance, including entomological surveillance targeting mosquito vectors. A scoping review was carried out to document the public health contributions of entomological surveillance of arboviruses of importance in Canada.

**Methods:**

The Ovid® and EBSCO platforms and the grey literature were searched to identify documents published between 2009 and 2023, in English or French, dealing with entomological surveillance of arboviruses of interest, conducted annually for human health purposes under the aegis of a government authority, with specified public health objectives and actions.

**Results:**

The 42 selected publications mainly reported two public health objectives of adult mosquito surveillance: early warning of viral circulation and assessment of the level of risk of human transmission. Recommended actions included clinical preparedness, risk communication, promotion of personal protection measures and vector control. The main objectives of immature mosquito surveillance were to identify sites with high larval densities, in order to reduce/eliminate them and target the application of larvicides.

**Conclusion:**

In a context of climate change favouring the spread of arboviruses, this study highlights the potential public health contributions of regular entomological surveillance of endemic arboviruses of importance in Canada. It helps support concrete actions to protect the health of the population from the risks of arboviral transmission.

## Introduction

Increased ambient temperatures and variability in precipitation patterns associated with climate change are conducive to an expansion in the geographic range of mosquito vectors of arboviruses endemic to Canada, an increase in their local abundance and a reduction in the extrinsic incubation period, enabling them to become infectious earlier ((1,2)). This greater dispersion would contribute to an increased risk of human transmission, particularly of West Nile virus (WNV), Eastern equine encephalitis virus (EEEV) and California serogroup viruses (CSV) ((1–3)).

These changes call for enhanced surveillance of such arboviruses to better assess the health risks to the Canadian population ((1)) and target interventions more effectively. To the best of our knowledge, no synthesis of the public health objectives of entomological surveillance of mosquito-borne arboviruses has been published in Canada.

The aim of this scoping review was to document, as comprehensively as possible, the public health objectives of the entomological component of surveillance for arboviruses of interest, namely WNV, EEEV, Cache Valley virus (CVV) and the CSVs, including Jamestown Canyon virus (JCV) and Snowshoe Hare virus (SSHV). In other words, the aim was to show how entomological surveillance data can be used to support various actions designed to protect the population from the risk of arbovirus transmission. The purpose of this study is therefore to support thinking on the potential of surveillance of mosquito vectors of these arboviruses of importance in Canada by examining the relevance of such surveillance to concrete action by the authorities concerned, including the implementation of appropriate preventive measures and vector control.

## Methods

### Search strategy

A scoping review was conducted based on the methodological framework suggested by Arksey and O'Malley ((4)) and improved by Levac *et al.* ((5)). Its specific aims were 1) to synthesize the public health objectives targeted by entomological surveillance of mosquito-borne arboviruses under different arboviral transmission scenarios as detailed below and 2) to describe how the resulting data can help support actions to protect the population. The public health objective implies, in fact, that the entomological surveillances reported in the literature are carried out with the aim of supporting concrete actions.

The research question was: "What are the contributions of the entomological component in the surveillance of WNV, EEEV, CVV and the CSVs, including JCV and SSHV, in a context of climate change?". For this research, the term "surveillance" refers to any process of ongoing data collection, carried out under the aegis of a governmental authority, particularly a public health authority, in order to guide its decisions, policies and responses (6). The research question identified three major concepts that were combined as follows: "arboviruses transmitted by mosquitoes," "surveillance" and "mosquito vectors."

For each of these major concepts, a list of synonymous keywords was drawn up for searching the bibliographic databases of the Ovid® (Embase, Global Health and Medline®) and EBSCO (CINAHL® Complete, Environment Complete and GreenFILE) platforms, as well as CAB Abstracts (CABI), Engineering Village, Pascal and Francis, PubMed and Web of Science. No geographical restrictions were applied and the literature search covered the period from 2009 to 2023. A complementary search was carried out in the grey literature for the same time interval. It considered mainly Google and Google Scholar search engines, in addition to grey literature resources, including government websites, notably those of health agencies in Canadian provinces and US states, among which are those bordering Canada.

Relevant publications were selected initially by evaluating titles and abstracts, and then by reading the full text, where necessary. Inclusion and exclusion criteria required that publications 1) be written in English or French; 2) deal with entomological surveillance for human health purposes of our arboviruses of interest with field collection of mosquitoes; 3) be regularly conducted each year during the mosquito season and initiated, supervised, requested, required or supported by one or more government entity(ies); and 4) have explicit public health objectives and possible subsequent actions implemented or recommended. Publications dealing, for example, with pure research activities, such as the advancement of knowledge on the ecology of mosquito vectors or trapping techniques, without mentioning public health objectives/actions, were excluded.

### Descriptive knowledge synthesis

A summary table was developed to report relevant data extracted from the selected publications. These data include the arboviruses and developmental stages targeted, the epidemiological situation during which entomological surveillance was carried out (i.e., no human cases, sporadic, endemic or epidemic human cases), the public health objectives targeted by this surveillance and the subsequent actions that can derive from the entomological data obtained.

The objectives identified were then classified according to four types of arboviral transmission scenarios based on the epidemiological situations described in the literature:

• **No arboviral transmission:** No human cases reported, no apparent arboviral transmission to the human population and low or unknown levels among reservoir hosts

• **Sporadic:** Human cases reported anecdotally, arboviral transmission considered sporadic, low level in the human population and among reservoir hosts

• **Endemic:** Human cases reported on a recurrent basis with no sign of sudden or rapid increase, arboviral transmission considered to be persistent in the human population and among reservoir hosts

• **Epidemic:** Sudden and rapid increase in human cases, arboviral transmission considered high and persistent in the human population and among reservoir hosts

## Results

**Figure 1:**
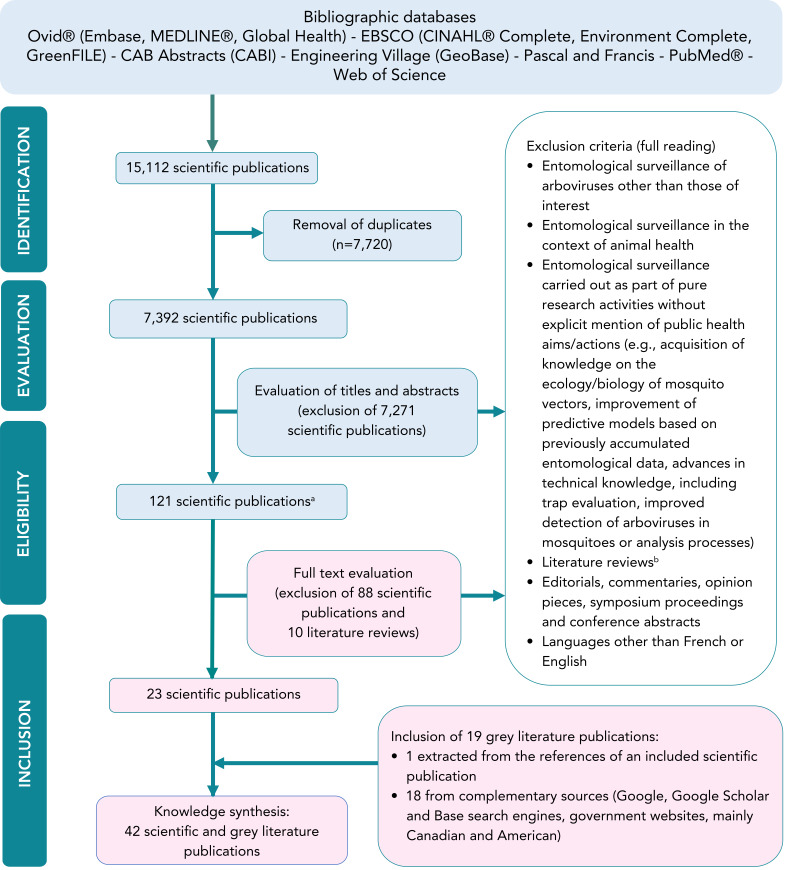
Flow chart illustrating the various stages in the search for and selection of relevant publications^a,b^ ^a^ The 121 articles retained following title and abstract evaluation included 111 to be assessed for eligibility by full text review and 10 literature reviews in an attempt to find any relevant publications not detected in the bibliographic databases ^b^ Literature reviews were excluded after extracting 13 scientific publications which were not retained after reading of the full text Restrictions used: from 2009 to 2023; no geographical restrictions were applied for document searches

**Figure 1** shows a flow chart illustrating the search and selection of relevant publications. Bibliographic database queries yielded 15,112 results. After removing duplicates, 7,392 scientific publications were evaluated by title and abstract. Only 121 were finally screened for eligibility by full text reading, including 10 literature reviews. These reviews were initially retained in an attempt to find relevant references not detected in the bibliographic databases. They were afterwards excluded. Of these 121 scientific publications, 23 were deemed eligible. Consultation of grey literature sources led to the addition of 18 documents, mainly recent entomological surveillance and intervention plans or reports. A grey literature document was also found in one of the accepted scientific articles. A total of 42 publications were included in the knowledge synthesis. The scientific articles are mainly from European countries, while the grey literature is more from North America. Most of these publications focused on two or even three mosquito-borne arboviruses. However, the vast majority of cases involved WNV (n=38), with the remainder involving EEEV (n=12) and JCV (n=3). No relevant documents on SSHV or CVV were identified (n=0).

A summary of the public health objectives of entomological surveillance of arboviruses of interest is presented in **Table 1** (adult mosquitoes) and **Table 2** (immature forms of mosquitoes). Surveillance of these different developmental stages is usually carried out concomitantly in order to consider the entire vector lifecycle ((7–20)). The two tables also include the number of publications reporting on each of the public health objectives, the arboviral transmission scenarios concerned and the arbovirus(es) of interest targeted per scenario, as well as examples of entomological indicators that help to achieve the targeted objectives.

**Table 1:** Public health objectives of entomological surveillance of arboviruses of interest by arboviral transmission scenario of adult mosquito surveillance

Public health objectives		n^a^	Arboviral transmission scenarios concerned				Example of entomological indicators used
			None	Sporadic	Endemic	Epidemic	
Early warning of viral circulation before the first human cases appear		20	WNV	WNV	WNV, EEEV, JCV	-	First positive mosquito pools for one and/or another of the arboviruses ((7,9–11,14,18,19,21–33))
Human risk assessment	Assessing the level of risk of human transmission^b^	22	WNV	WNV	WNV, EEEV, JCV	-	Spatiotemporal distribution and abundance of mosquitoes by identified species, number of mosquitoes per trap, number of positive mosquito pools, number of traps with positive mosquitoes, species type of positive mosquitoes (more ornithophilic or more that feed on mammals, including mosquitoes that feed on humans), number of weeks with positive mosquito pools, infection rate^c^, vector index^d^ ((7–19,21,32,34–40))
	Mapping levels of viral circulation intensity	3	-	-	WNV	-	Mosquito infection rate (maximum likelihood estimation and minimum infection rate)^c^ (22–24)
	Predicting an outbreak of human cases	2	-	-	WNV	-	Proportion of positive mosquito pools, minimum infection rate, vector index ((22,33))
Assessing resistance to insecticides used in vector control		7	-	-	WNV, EEEV, JCV	WNV	Mosquito abundance before and after insecticide treatment, presence and frequency of mutation genes ((9,11,16,17,20,41,42))
Real-time monitoring and support for efforts to reduce human transmission		3	-	-	-	WNV, EEEV	Mosquito abundance, number of positive mosquito pools, vector index, minimum infection rate ((43–45))
Contribution to the declaration of a health emergency linked to arboviruses		1	-	-	WNV, EEEV	-	Proportion of positive mosquito pools (health emergency declared as soon as 10% of bridge vector^e^ mosquito pools tested positive for WNV or EEEV) ((14))
Controlling the spread of the *Culex* population from flooded areas^f^		1	-	-	WNV	-	Abundance of *Culex* species per trap ((35))
Update of the list of potential vector species		1	-	-	WNV	-	Adult mosquito abundance by identified species, minimum infection rate (46)
Documentation of WNV transmission and overwintering mechanisms in competent vectors^g^		1	-	-	WNV	-	Presence of WNV in hibernating mosquitoes ((9))
Documenting the intensity of viral circulation during an epidemic year at international airports^h^		1	-	-	-	WNV	Mosquito abundance by identified species, minimum infection rate ((47)
Warning of a potentially increased risk of arboviral transmission for next year's mosquito season^i^		1	-	-	-	WNV	Mosquito abundance by identified species, minimum infection rate ((48)

Abbreviations: EEEV, Eastern equine encephalitis virus; JCV, Jamestown Canyon virus; WNV, West Nile virus; -, not applicable

^a^ n is the number of publications having documented the public health objective. The same publication could report on several public health objectives. The sum of these numbers is therefore greater than 42

^b^ Also known as the probability of locally acquired human diseases in the states of Massachusetts and Rhode Island, or the probability of human illness in the state of New Hampshire. Risk levels for human transmission are generally classified as low, moderate, high or very high

^c^ The infection rate corresponds to the number of infected mosquitoes per 1,000 tested. It can be expressed using two indicators: maximum likelihood estimation, which assumes that one or more mosquitoes are infected in a pool tested positive for the targeted arbovirus, or minimum infection rate, which is a simple approximation of the prevalence of infected mosquitoes, since it assumes that only one mosquito is positive in each positive pool

^d^ The vector index, or risk index, is the estimated proportion of infected mosquitoes of a particular species in a specific area. It corresponds to the product of the number of mosquitoes collected and their infection rate

^e^ A bridge vector mosquito is capable of carrying the pathogen and transmitting it to another species (including humans) other than the one involved in the enzootic cycle

^f^ *Culex* mosquitoes are strongly influenced by temperature, precipitation and humidity

^g^ Based on the hypothesis that some *Culex pipiens* may survive the winter as adults while infected. Adult mosquitoes are collected during the off-season (from November to the end of March)

^h^ During epidemic years, the ecological habitats of airports can favour WNV transmission and increase the risk of mosquitoes and/or viruses spreading to non-endemic regions

^i^ This higher risk is associated with a milder winter combined with the ability of the main infected mosquito vectors to spend the winter season in the geographical area where entomological surveillance takes place

**Table 2:** Public health objectives of entomological surveillance of arboviruses of interest by arboviral transmission scenario of immature mosquito surveillance

Public health objectives	n^a^	Arboviral transmission scenarios concerned				Example of entomological indicators used
		None	Sporadic	Endemic	Epidemic	
Identifying larval breeding sites^b^ and determining high larval density areas	16	WNV	WNV	WNV, EEEV, JCV	-	Presence of eggs, larvae and pupae; abundance (or density) by identified species and developmental stage^c^ (7–20,32,37)
Mapping of breeding sites^d^	6	-	-	WNV, EEEV, JCV	-	Presence of breeding sites ((12,15,17–20))

Abbreviations: EEEV, Eastern equine encephalitis virus; JCV, Jamestown Canyon virus; WNV, West Nile virus; -, not applicable

^a^ n is the number of publications having documented the public health objective. The same publication could report on both of the public health objectives described. The sum of these numbers is therefore greater than 16

^b^ The location of artificial and natural breeding sites is linked to the specific ecology of each mosquito vector

^c^ The abundance of immature mosquitoes is an early indicator of the density of the future adult population

^d^ The collection and examination of topographical maps, aerial photographs, geographic information systems (GIS) technology and local expertise can be used to map breeding sites

The majority of public health objectives identified in the consulted literature concern the scenario of arboviral transmission at endemic level. Those most documented for adult mosquito surveillance, and common to WNV, EEEV and JCV, are:

• Early warning of viral circulation (n=20) before the first human cases appear and thanks to the first positive mosquito vectors pools for one and/or another of the arboviruses under surveillance.

• Assessment of the level of risk of human transmission (n=22) based on, among others, entomological indicators such as mosquito abundance, infection rate of arbovirus in mosquito population and vector index. These risk levels are generally described as low, moderate, high or very high.

The main objectives reported for surveillance of immature forms of the vectors of one and/or another of these three arboviruses are the identification of artificial and natural breeding sites and the determination of areas with high larval densities (n=16).

**Table 3** and **Table 4** summarize the main public health actions that can derive from adult and immature mosquito surveillance data, respectively. These actions are presented for each surveillance objective. For adult forms, they include:

**Table 3:** Public health actions that can derive from adult mosquito surveillance data

Surveillance objectives		Public health actions			
		Clinical preparedness^a^	Risk communication^b^	Ongoing awareness/education campaigns^c^	Vector control^d^
Early warning of viral circulation before the first human cases appear		X^e^	X^e^	X^e^	X^e^
Human risk assessment	Assessment of the level of risk of human transmission	X^e^	X^e^	X^e^	X^e^
	Mapping levels of viral circulation intensity	-	-	-	X^e^
	Predicting an outbreak of human cases	-	-	-	X^e^
Evaluation of resistance to insecticides used in vector control^f^		-	-	-	X^e^
Real-time monitoring and support for efforts to reduce human epidemic transmission^g^		X^e^	X^e^	X^e^	X^e^
Contribution to the declaration of a health emergency linked to arboviruses^h^		-	X^e^	X^e^	X^e^
Controlling the spread of the *Culex* population from flooded areas		-	X^e^	X^e^	X^e^
Update of the list of potential vector species^f^		-	-	-	X^e^
Documentation of WNV transmission and overwintering mechanisms in competent vectors		-	-	-	X^e^
Documenting the intensity of viral circulation during an epidemic year at international airports		-	-	-	X^e^
Warning of a potentially increased risk of arboviral transmission for next year's mosquito season^i^		-	X^e^	X^e^	-

Abbreviations: WNV, West Nile virus; -, not applicable

^a^ Includes increased vigilance in the recognition and diagnosis of arboviruses, an expansion of technical laboratory resources for confirmatory testing of human cases, the strengthening of veterinary surveillance and the activation of the procedure guaranteeing the safety of blood transfusions

^b^ Includes real-time, regular and constantly updated communication of the levels of risk of human transmission to local authorities, health care providers, the media (e.g., publication of national press releases) and the general public

^c^ Includes awareness-raising and ongoing education of human healthcare providers and the general population, especially those at risk, on personal protection measures (e.g., long-sleeved clothing, mosquito nets, use of repellents) and source reduction by eliminating peridomestic and urban stagnant water (e.g., emptying of artificial containers, recovery of used tires, swimming pool maintenance), while multiplying communication platforms (e.g., websites of health authorities and other relevant government entities, press releases in local newspapers, interviews on various cable channels, social networks, information leaflets in different languages in schools and community organizations, workshops in retirement homes) to increase outreach efforts

^d^ Includes source reduction, ground and/or aerial application of larvicides and even of adulticides, when the level of risk of human transmission have been deemed high or even critical

^e^ For each of the adult mosquito surveillance objectives, the coloured cells designate the public health actions that can be derived from the surveillance data

^f^ Public health action involves updating vector control programs where necessary

^g^ Public health action consists of an increase in confirmatory testing of human cases and an intensification of risk communication strategies, personal protection awareness/education campaigns and vector control in the most at-risk areas

^h^ Public health actions can include the creation of a panel of experts and the setting up of an emergency operations centre for coordinated, faster and more effective responses

^i^ Public health actions consist of a sustained, ongoing education/awareness campaign for the general population and risk communication to public health authorities in neighbouring states and regions

**Table 4:** Public health actions that can derive from immature mosquito surveillance data

Surveillance objectives	Public health actions			
	Source reduction^a^	Targeted larvicidal treatments^b^	Evaluation of larvicidal treatments^c^	Real-time monitoring of larvicide deployment^d^
Identifying larval breeding sites and determining high larval density areas	X^e^	X^e^	X^e^	-
Mapping of breeding sites^f^	-	-	-	X^e^

^a^ Includes elimination of stagnant water (e.g., percolation, recirculation, drainage), vegetation management (e.g., controlling algae growth) and saltmarsh water management

^b^ Involves targeting priority areas with high larval densities

^c^ Includes evaluation of duration and efficacy of larvicidal treatments

^d^ Involves checking the suitability of larvicidal treatments in priority areas with high larval densities

^e^ For each of the immature mosquito surveillance objectives, the coloured cells designate the public health actions that can be derived from the surveillance data

^f^ Enables improved surveillance of breeding sites for the following year's mosquito season by allowing the responsible authorities to decide where to concentrate this surveillance

• Clinical preparedness to strengthen human surveillance, particularly through greater vigilance in recognizing and diagnosing illnesses linked to these three arboviruses, as well as increased laboratory resources for confirmatory testing of human cases.

• Real-time risk communication by the responsible authorities to local authorities, healthcare providers, the media and the general public.

• Ongoing education/awareness campaigns, using a variety of communication channels to increase outreach efforts, aimed at the general population and healthcare professionals. These campaigns focus mainly on personal protection measures (e.g., long-sleeved clothing, mosquito nets, use of repellents) and participation in source reduction efforts by eliminating peridomestic stagnant water (e.g., emptying artificial containers, recovering used tires, swimming pool maintenance).

• Vector control, including the ground-based and/or aerial application of larvicidal treatments or even of adulticides, when the level of risk of human transmission is deemed high or critical.

Public health actions guided by data from immature forms surveillance essentially include source reduction aimed at eliminating/reducing natural and artificial larval breeding sites (e.g., elimination of stagnant water and vegetation management), targeted larvicidal treatments focusing on areas of high larval density and evaluation of the effectiveness of such treatments.

## Discussion

### Entomological surveillance of mosquito-borne arboviruses: a valuable contribution to public health

This literature review has documented the public health objectives that can be achieved through entomological surveillance of arboviruses of interest, as well as the subsequent actions that can derive from the resulting data. These objectives were reported by developmental stage monitored, i.e., adult and immature mosquitoes, and by arboviral transmission scenario. This strategic breakdown of public health objectives/actions according to various scenarios offers some avenues for reflection to carry out mosquito surveillance. Any authority concerned could then, according to its priorities, opt for the appropriate objective(s) in line with the arboviral transmission scenario prevailing in the region targeted for entomological surveillance of mosquito-borne arboviruses.

The major finding of this study is that mosquito surveillance can help support the implementation of actions to protect human health from the risk of arboviral transmission. Real-time exploitation of entomological data from adult mosquito surveillance provides useful and rapid information to the authorities concerned by contributing to early warning of viral circulation and by helping to assess the level of risk of human transmission to support more prompt and informed management. The aim is to decrease arboviral transmission and limit human cases by implementing a range of preventive public health actions, including vector control. The latter intends to reduce the abundance of infected or potentially infected vectors, thereby lowering the environmental viral load.

Early warning of viral circulation is based on the detection of the first positive mosquito pools for the arboviruses under surveillance, which usually occur a few days or even several weeks before human cases appear ((9,22,23,26,30,31,42)). In New York City, for example, data collected between 2000 and 2022 showed that WNV was detected in mosquitoes weeks before any risk of human transmission became significant ((9)). The main purpose of this alert is to rapidly initiate clinical preparedness activities, as described above, risk communication, coordination and training of local health officials and personnel involved in entomological surveillance, as well as the development of materials for public awareness/education campaigns on preventive measures. Early warning also enables vector control to be initiated, including source reduction, to limit the spread of infected mosquitoes to densely populated areas ((7–9,14,17–19,21–33))

The level of risk of human transmission is assessed throughout the mosquito season using real-time entomological data from the current year, often combined with those from previous years ((10–13,16–18)). These data are most often also combined with other parameters, as no single indicator can provide an accurate measure of risk ((10,18)). These parameters include ((9,11,13,14,16–18,35)):

• Immature mosquito surveillance data, including type and location of breeding sites and their proximity to the human population at risk and larval abundance

• Human and animal surveillance data (wild birds, chickens, horses, etc.)

• The time of year

• Current and projected local weather conditions (degree-day accumulation, precipitation, wind speed, etc.)

• The density of the human population at risk, particularly those close to larval breeding sites

The use of entomological data to estimate the level of risk of human transmission is justified by the statistically positive correlation between mosquito abundance, vector index and/or mosquito infection rate, on the one hand, and the number of human cases, on the other. This correlation has been well documented for WNV in Canada (49,50) and elsewhere in the world ((33,44,51,52)).

This risk level assessment helps guide the rapid implementation and gradual, targeted and proportionate intensification of public health actions, including regular risk communication and updates, education/awareness-raising through public outreach campaign on preventive measures and vector control. The focus is put on a gradual reinforcement of personal protection measures for humans and source reduction measures, or even a possible restriction of outdoor activities to decrease exposure risks (10,13,16–18,34–38). The states of Massachusetts ((10)), Vermont (17), New Hampshire ((18)) and Rhode Island (38) have, in fact, developed guidelines revealing the entomological data, combined or not with other parameters, that define levels of risk of human transmission and the subsequent public health responses.

Outbreak risk assessment is generally based on the vector index, which predicts an increase in the number of human cases over the following two to three weeks ((33,43,50)). This predictive effort can be used to guide vector control strategies in order to prioritize areas identified as being most at risk ((32,33)).

Other public health objectives were identified in the consulted literature. Although few publications have reported on them, they remain relevant. One example is the assessment of resistance to insecticides used in vector control, which is essential for evidence-based strategy revision. Another example would be the contribution to the declaration of a health emergency linked to arboviruses, prompting the creation of a panel of experts (epidemiologists, veterinarians, vector control experts, biologists, local representatives) and the setting up of an emergency operations centre for coordinated, faster and more effective public health interventions ((14)).

Finally, surveillance of immature mosquitoes is essential, as it enables targeted larvicidal treatments to help reduce the adult mosquito population, particularly when the level of risk of human transmission is deemed high ((11,17)). Detailed documentation of the presence and abundance of immature mosquitoes, the developmental stages treated by larvicides, the size of breeding sites and the effectiveness of vector control is considered of great value in continuously estimating the likely size of future adult mosquito populations ((10,14,18)).

### Optimal conditions for more effective entomological surveillance

The literature review also identified relevant information on the optimal conditions for strengthening the efficiency of entomological surveillance strategies for adult mosquitoes to achieve the main public health objectives documented.

### As an early warning tool for viral circulation

As reported for WNV, early warning of viral circulation depends on certain operational modalities, in particular:

• Intensive trapping to increase the number of mosquitoes to be collected and tested, as this parameter is crucial for the sensitivity of early arbovirus detection ((21,28,30)). This condition implies a substantial number of mosquito traps located in "hot spots," selected according to a multifactorial approach (e.g., presence of wetlands and other water bodies, human population density, meteorological parameters) (21,30). Thomas-Bachli *et al.* ((53)) demonstrated that increasing the number of traps in Ontario, combined with shifting their locations to areas where WNV had been detected in previous years, improved detection times for arbovirus in mosquitoes, which became similar to or even shorter than those associated with dead corvid surveillance ((53)). A judicious choice of the type of mosquito traps and their wide deployment also enhances the ability of entomological surveillance to provide early warning of viral circulation ((25)).

• Rapid acquisition, ideally within a few days, of results of WNV screening in mosquito population ((9,26,27)).

• Maintain regular surveillance on an annual basis, preferably from May until the end of the mosquito season (usually late September), in order to improve strategy and refine early detection capabilities and sensitivity ((24)).

• Collaboration between veterinary and human health services, as well as between medical entomologists and ornithologists, in addition to coordination and data management at national, regional and local levels ((24)).

• Regular updating of the entomological surveillance program in line with available data (results from the previous year and those obtained from research studies) and funding opportunities ((24,27)).

### As a tool for assessing the level of risk of human transmission

The development of appropriate models for assessing human transmission risk levels, using entomological data, also requires ongoing surveillance carried out every year during the mosquito season. It also calls for rapid processing and analysis of entomological data, so that the necessary preventive measures can be implemented without delay ((27,50)). It is also strongly recommended that monitoring programs include permanent traps placed at fixed stations, with a long-term perspective, in order to develop a historical baseline for detecting spatiotemporal trends in mosquito abundance and arbovirus prevalence within their populations. In fact, the assessment of the level of risk of human transmission generally incorporates the results of mosquito surveillance from previous years. The constant accumulation of entomological data, year after year, also offers the opportunity to improve the robustness of predictive models for more accurate estimates of human risk, including the occurrence of outbreaks (10,14,18,19,24,27). In New York City, comprehensive vector and human surveillance data collected over the years 2006 to 2022 enabled health authorities to develop a more sensitive protocol for assessing the level of WNV activity and human disease risk across the entire city ((9)). In Massachusetts, routine seasonal data collection over a period of several years also considerably improved the accuracy of assessing the level of risk of human transmission at the municipal level ((10)).

Karki *et al.* ((54)) have demonstrated, for the state of Illinois, that the power of predictive models based on vector index increases in regions with abundant entomological data. The authors also highlighted their usefulness, when collected over the long term, for developing risk assessments at specific times and in specific regions to guide an appropriate public health response ((54)).

On the other hand, Kilpatrick and Pape (50) warned against the loss of data resulting from discontinuous entomological surveillance. Thus, a pronounced decline in the predictive power of the models used is expected in the absence of arbovirus prevalence and mosquito abundance data for the surveillance year. Moreover, the decline in predictive power is exacerbated by delays in processing and analyzing entomological data (50).

Finally, it should be noted that the consulted literature also highlighted the importance of sharing roles and responsibilities for the smooth running of entomological surveillance programs, including the operational aspect (e.g., selection of locations and installation of mosquito traps), the assessment of the level of risk of human transmission and the resulting public health responses, particularly the application of larvicides and adulticides. Mosquito surveillance programs can therefore involve various levels of health authorities, as well as other government bodies, notably from the agricultural and environmental sectors, and local administrations such as municipalities ((14,16,17,19,38,45)).

## Strengths and limitations

The main strength of this work lies in the inclusion of publications dealing with entomological surveillance carried out annually, on a regular and uninterrupted basis, while involving government authorities at the national, regional or local level. This approach lent greater weight to the public health objectives of this surveillance and subsequent actions documented in the literature. Furthermore, the surveillance programs described are still underway in the countries/regions concerned, as they are considered to be relevant, which further strengthens this knowledge synthesis. However, this review cannot claim to be exhaustive, as additional results could have been identified with less stringent inclusion criteria. In addition, there have been few publications on EEEV and the CSVs and no relevant literature on SSHV or CVV has been identified. These factors could make it harder to infer about these arboviruses, which, however, are likely to grow in importance in the coming years. Beyond these limitations, we are confident in the relevance and significance of the results obtained and believe that this review remains a first description of the synthesis of the objectives of entomological surveillance of arboviruses of public health interest and endemic to Canada.

## Conclusion

In a context of climate change conducive to the spread of arboviruses, this knowledge synthesis supports the usefulness and relevance of entomological surveillance of arboviruses of interest in Canada, namely WNV, EEEV and JCV. Its contribution to public health is nevertheless grounded in a regular annual deployment during the mosquito season, according to the objectives pursued by the authorities concerned, while using a judicious number and locations of mosquito traps. For optimum benefit, it is also vital that entomological data are analyzed and shared rapidly to support effective actions, integrating clinical preparedness, real-time and ongoing risk communication as well as timely implementation of preventive measures. Entomological surveillance of arboviruses of public health importance should be maintained and strengthened, taking into consideration expected changes, due to climate variations, in mosquito populations and the diseases they carry in Canada.

## References

[1] Ludwig A, Zheng H, Vrbova L, Drebot MA, Iranpour M, Lindsay LR. Increased risk of endemic mosquito-borne diseases in Canada due to climate change. Can Commun Dis Rep 2019;45(4):91–7. 10.14745/ccdr.v45i04a03 31285698 PMC6587694

[2] Ogden NH, Gachon P. Climate change and infectious diseases: what can we expect? Can Commun Dis Rep 2019;45(4):76–80. 10.14745/ccdr.v45i04a01 31285696 PMC6587697

[3] Rosenkrantz L; National Collaborating Centre for Environmental Health. Impacts of Canada's changing climate on West Nile Virus vectors. Vancouver, BC: NCCEH; 2022. https://ncceh.ca/sites/default/files/Mosquito_EvidenceReview_Nov1_EN.pdf

[4] Arksey H, O’Malley L. Scoping studies: towards a methodological framework. International journal of social research methodology. Int J Soc Res Methodol 2005;8(1):19–32. 10.1080/1364557032000119616

[5] Levac D, Colquhoun H, O’Brien KK. Scoping studies: advancing the methodology. Implement Sci 2010;5(69):69. 10.1186/1748-5908-5-69 20854677 PMC2954944

[6] Lussier MT, Richard C, Bennett TL, Williamson T, Nagpurkar A. Surveillance or research: what's in a name? Can Fam Physician 2012;58(1):117. https://.ncbi.nlm.nih.gov/22267632/PMC3264028 22267632

[7] Osório HC, Zé-Zé L, Amaro F, Alves MJ. Mosquito surveillance for prevention and control of emerging mosquito-borne diseases in Portugal - 2008-2014. Int J Environ Res Public Health 2014;11(11):11583–96. 10.3390/ijerph111111583 25396768 PMC4245631

[8] Medlock JM, Guillem R, Johnston C, Gandy S, Findlay-Wilson S, Desoisa K, Schaffner F, Vaux AG. The first 6 years of surveillance of Aedes albopictus (Diptera: Culicidae) in Gibraltar. J Eur Mosq Control Assoc 2022;40:23–35. 10.52004/JEMCA2022.0001

[9] Bajwa W, Slavinski S, Shah Z, Zhou L, Herbert V. Comprehensive Mosquito Surveillance and Control Plan. New York City Department of Health and Mental Hygiene; 2023. p. 41. https://www.nyc.gov/assets/doh/downloads/pdf/wnv/2023/wnvplan2023.pdf

[10] Brown CM, DeMaria A Jr, Gallagher GR, Osborne M, Stinson C, Smole S, Werner BG. Massachusetts Arbovirus Surveillance and Response Plan. Bureau of infectious Disease and Laboratory Sciences, Massachusetts Department of Public Health; 2023. https://www.mass.gov/lists/arbovirus-surveillance-plan-and-historical-data

[11] California Department of Public Health. California Mosquito-borne Virus Surveillance & Response Plan. Mosquito & Vector Control Association of California; 2023. https://westnile.ca.gov/pdfs/CAMosquitoSurveillanceResponsePlan.pdf

[12] City of Fort Collins. West Nile Virus Program Manual. Fort Collins, CO: City of Fort Collins; 2014. https://www.fcgov.com/westnile/pdf/wnv_program_manual.pdf

[13] Government of Saskatchewan. West Nile Virus (WNV). Saskatoon, SK: Government of Saskatchewan; 2023. https://www.saskatchewan.ca/residents/health/diseases-and-conditions/west-nile-virus

[14] Maine Department of Health and Human Services/Maine Center for Disease Control and Prevention. Arboviral (Mosquito-Borne) Illness, Surveillance, Prevention, and Response Guidance for Maine Towns and Communities. Augusta, ME: Maine DHHS; 2021. https://www.maine.gov/dhhs/mecdc/infectious-disease/epi/vector-borne/documents/2022-Arbo-Plan.pdf

[15] Manitoba Health. West Nile Virus Program 2023: Planning Documents for Municipalities: I. Provincial West Nile Viruses Program Information. Winnipeg, MB: Manitoba Health; 2023. https://www.gov.mb.ca/health/wnv/docs/wnv_program_information_2023.pdf

[16] Ontario Ministry of Health. West Nile Virus Preparedness and Prevention Plan. Toronto, ON: King's Printer for Ontario; 2023. https://files.ontario.ca/moh-ophs-ref-west-nile-virus_plan-2023-en.pdf

[17] Vermont Agency of Agriculture, Food, and Markets/Vermont Department of Public Health. State of Vermont. Arbovirus Surveillance and Response Plan. Vermont, NH: Vermont Department of Public Health; 2022. https://www.healthvermont.gov/sites/default/files/documents/pdf/HS-ID-Arbovirus-Surveillance-Response-Plan.pdf

[18] State of New Hampshire. Arboviral Illness Surveillance, Prevention and Response Plan. Department of Health and Human Services, Division of Public Health Service; 2023. https://www.dhhs.nh.gov/sites/g/files/ehbemt476/files/documents/2021-11/arboviralresponse.pdf

[19] New York State Department of Health. Mosquito Borne Illness Surveillance & Response Plan. New York, NY: New York State Department of Health; 2012. https://www.health.ny.gov/diseases/west_nile_virus/docs/2012_mosquito_borne_illness_surveillance_and_response_plan.pdf

[20] Saginaw County Mosquito Abatement Comission. Surveillance Biology. Saginaw County, MI: Saginaw County Mosquito Abatement Comission; 2024. https://www.saginawmosquito.com/programs/surveillance

[21] Pautasso A, Radaelli MC, Ballardini M, Francese DR, Verna F, Modesto P, Grattarola C, Desiato R, Bertolini S, Vitale N, Ferrari A, Rossini I, Accorsi A, Mosca A, Monaco F, Savini G, Prearo M, Mignone W, Chiavacci L, Casalone C. Detection of West Nile and Usutu Viruses in Italian Free Areas: Entomological Surveillance in Piemonte and Liguria Regions, 2014. Vector Borne Zoonotic Dis 2016;16(4):292–4. 10.1089/vbz.2015.1851 26862776

[22] Calzolari M, Angelini P, Bolzoni L, Bonilauri P, Cagarelli R, Canziani S, Cereda D, Cerioli MP, Chiari M, Galletti G, Moirano G, Tamba M, Torri D, Trogu T, Albieri A, Bellini R, Lelli D. Enhanced West Nile Virus Circulation in the Emilia-Romagna and Lombardy Regions (Northern Italy) in 2018 Detected by Entomological Surveillance. Front Vet Sci 2020;7:243. 10.3389/fvets.2020.00243 32432132 PMC7214930

[23] Petrović T, Šekler M, Petrić D, Lazić S, Debeljak Z, Vidanović D, Ignjatović Ćupina A, Lazić G, Lupulović D, Kolarević M, Plavšić B. Methodology and results of integrated WNV surveillance programmes in Serbia. PLoS One 2018;13(4):e0195439. 10.1371/journal.pone.0195439 29624622 PMC5889191

[24] Petrović T, Šekler M, Petrić D, Vidanović D, Debeljak Z, Lazić G, Lupulović D, Kavran M, Samojlović M, Ignjatović Ćupina A, Tešović B, Lazić S, Kolarević M, Labus T, Djurić B. Intensive West Nile Virus Circulation in Serbia in 2018-Results of Integrated Surveillance Program. Pathogens 2021;10(10):1294. 10.3390/pathogens10101294 34684243 PMC8540029

[25] Verna F, Modesto P, Radaelli MC, Francese DR, Monaci E, Desiato R, Grattarola C, Peletto S, Mosca A, Savini G, Chianese R, Demicheli V, Prearo M, Chiavacci L, Pautasso A, Casalone C. Control of Mosquito-Borne Diseases in Northwestern Italy: Preparedness from One Season to the Next. Vector Borne Zoonotic Dis 2017;17(5):331–9. 10.1089/vbz.2016.2047 28437184

[26] Papa A, Gewehr S, Tsioka K, Kalaitzopoulou S, Pappa S, Mourelatos S. Detection of flaviviruses and alphaviruses in mosquitoes in Central Macedonia, Greece, 2018. Acta Trop 2020;202:105278. 10.1016/j.actatropica.2019.105278 31756306

[27] Patsoula E, Vakali A, Balatsos G, Pervanidou D, Beleri S, Tegos N, Baka A, Spanakos G, Georgakopoulou T, Tserkezou P, Van Bortel W, Zeller H, Menounos P, Kremastinou J, Hadjichristodoulou C. West Nile Virus Circulation in Mosquitoes in Greece (2010-2013). BioMed Res Int 2016;2016:2450682. 10.1155/2016/2450682 27294111 PMC4880692

[28] Tsioka K, Gewehr S, Pappa S, Kalaitzopoulou S, Stoikou K, Mourelatos S, Papa A. West Nile Virus in *Culex* Mosquitoes in Central Macedonia, Greece, 2022. Viruses 2023;15(1):224. 10.3390/v15010224 36680264 PMC9863787

[29] Alba A, Allepuz A, Napp S, Soler M, Selga I, Aranda C, Casal J, Pages N, Hayes EB, Busquets N. Ecological surveillance for West Nile in Catalonia (Spain), learning from a five-year period of follow-up. Zoonoses Public Health 2014;61(3):181–91. 10.1111/zph.12048 23590452

[30] Calzolari M, Bonilauri P, Bellini R, Albieri A, Defilippo F, Maioli G, Galletti G, Gelati A, Barbieri I, Tamba M, Lelli D, Carra E, Cordioli P, Angelini P, Dottori M. Evidence of simultaneous circulation of West Nile and Usutu viruses in mosquitoes sampled in Emilia-Romagna region (Italy) in 2009. PLoS One 2010;5(12):e14324. 10.1371/journal.pone.0014324 21179462 PMC3002278

[31] Calzolari M, Monaco F, Montarsi F, Bonilauri P, Ravagnan S, Bellini R, Cattoli G, Cordioli P, Cazzin S, Pinoni C, Marini V, Natalini S, Goffredo M, Angelini P, Russo F, Dottori M, Capell G, Savini G. New incursions of West Nile virus lineage 2 in Italy in 2013: the value of the entomological surveillance as early warning system. Vet Ital 2013;49(3):315–9. 10.12834/VetIt.1308.04 24002939

[32] Saginaw County Mosquito Abatement Commission. Annual Report. Saginaw County, MI: Saginaw County Mosquito Abatement Commission; 2022. https://www.saginawmosquito.com/news/publications/annualreport

[33] Gobbi F, Capelli G, Angheben A, Giobbia M, Conforto M, Franzetti M, Cattelan AM, Raise E, Rovere P, Mulatti P, Montarsi F, Drago A, Barzon L, Napoletano G, Zanella F, Pozza F, Russo F, Rosi P, Palù G, Bisoffi Z; Summer Fever Study Group. Human and entomological surveillance of West Nile fever, dengue and chikungunya in Veneto Region, Italy, 2010-2012. BMC Infect Dis 2014;14:60. 10.1186/1471-2334-14-60 24499011 PMC3922982

[34] City of Fort Collins. Program Response Guidelines to Mosquito-Borne Arboviral Activity. Fort Collins, CO: City of Fort Collins; 2018. https://www.fcgov.com/westnile/pdf/WNV_Response_Plan_March_2018.pdf?1568214777

[35] Kansas Department of Health and Environment. Arboviral Disease Surveillance. Kansas, KS: Kansas Department of Health and Environment; 2019. https://www.kdhe.ks.gov/DocumentCenter/View/23922/Arboviral-Surveillance-Report-2019-PDF

[36] Kansas Department of Health and Environment. West Nile Virus. Kansas, KS: Kansas Department of Health and Environment; 2023. https://www.kdhe.ks.gov/1519/West-Nile-Virus

[37] Ginsberg HS, Gettman A, Becker E, Bandyopadhyay AS, Lebrun RA. Environmental management of mosquito-borne viruses in Rhode Island. R I Med J (2013) 2013;96(7):37–41. http://www.rimed.org/rimedicaljournal/2013/07/2013-07-37-cont-mosquitos.pdf 23819140

[38] Rhode Island Department of Health. Guidelines for Phased Response to Eastern Equine Encephalitis (EEE) Surveillance Data. Division of Preparedness, Response, Infectious Disease, and EMS Center for Acute Infectious Disease Epidemiology. Rhode Island, RI: Rhode Island Department of Health; 2019. https://health.ri.gov/publications/guidelines/PhasedResponseToEEESurveillanceData.pdf

[39] Oliver J, Lukacik G, Kokas J, Campbell SR, Kramer LD, Sherwood JA, Howard JJ. Twenty years of surveillance for Eastern equine encephalitis virus in mosquitoes in New York State from 1993 to 2012. Parasit Vectors 2018;11(1):362. 10.1186/s13071-018-2950-1 29941031 PMC6019270

[40] Public Health Ontario. Why Is It Important to Monitor Ontario's Mosquitoes? Ottawa, ON: PHO; 2018. https://www.publichealthontario.ca/en/about/news/2017/monitor-mosquitoes

[41] Fotakis EA, Mavridis K, Kampouraki A, Balaska S, Tanti F, Vlachos G, Gewehr S, Mourelatos S, Papadakis A, Kavalou M, Nikolakakis D, Moisaki M, Kampanis N, Loumpounis M, Vontas J. Mosquito population structure, pathogen surveillance and insecticide resistance monitoring in urban regions of Crete, Greece. PLoS Negl Trop Dis 2022;16(2):e0010186. 10.1371/journal.pntd.0010186 35176020 PMC8890720

[42] Mavridis K, Fotakis EA, Kioulos I, Mpellou S, Konstantas S, Varela E, Gewehr S, Diamantopoulos V, Vontas J. Detection of West Nile Virus - Lineage 2 in Culex pipiens mosquitoes, associated with disease outbreak in Greece, 2017. Acta Trop 2018;182:64–8. 10.1016/j.actatropica.2018.02.024 29474832

[43] Kretschmer M, Ruberto I, Townsend J, Zabel K, Will J, Maldonado K, Busser N, Damian D, Dale AP. Unprecedented Outbreak of West Nile Virus - Maricopa County, Arizona, 2021. MMWR Morb Mortal Wkly Rep 2023;72(17):452–7. 10.15585/mmwr.mm7217a1 37104168

[44] Martinez D, Murray KO, Reyna M, Arafat RR, Gorena R, Shah UA, Debboun M. West Nile Virus Outbreak in Houston and Harris County, Texas, USA, 2014. Emerg Infect Dis 2017;23(8):1372–6. 10.3201/eid2308.170384 28726615 PMC5547786

[45] Quilliam DN, Gosciminski M, Bandy U. Eastern Equine Encephalitis Surveillance and Response, Rhode Island, 2019. R I Med J (2013) 2020;103(3):68–70. 32236168

[46] Noden BH, Coburn L, Wright R, Bradley K. An Updated Checklist of the Mosquitoes of Oklahoma Including New State Records and West Nile Virus Vectors, 2003-06. J Am Mosq Control Assoc 2015;31(4):336–45. 10.2987/moco-31-04-336-345.1 26675455

[47] Bakran-Lebl K, Camp JV, Kolodziejek J, Weidinger P, Hufnagl P, Cabal Rosel A, Zwickelstorfer A, Allerberger F, Nowotny N. Diversity of West Nile and Usutu virus strains in mosquitoes at an international airport in Austria. Transbound Emerg Dis 2022;69(4):2096–109. 10.1111/tbed.14198 34169666 PMC9540796

[48] Kemenesi G, Krtinić B, Milankov V, Kutas A, Dallos B, Oldal M, Somogyi N, Németh V, Bányai K, Jakab F. West Nile virus surveillance in mosquitoes, April to October 2013, Vojvodina province, Serbia: implications for the 2014 season. Euro Surveill 2014;19(16):20779. 10.2807/1560-7917.ES2014.19.16.20779 24786260

[49] Giordano BV, Kaur S, Hunter FF. West Nile virus in Ontario, Canada: A twelve-year analysis of human case prevalence, mosquito surveillance, and climate data. PLoS One 2017;12(8):e0183568. 10.1371/journal.pone.0183568 28829827 PMC5568768

[50] Albrecht L, Kaufeld KA. Investigating the impact of environmental factors on West Nile virus human case prediction in Ontario, Canada. Front Public Health 2023;11:1100543. 10.3389/fpubh.2023.1100543 36875397 PMC9981635

[51] Bolling BG, Barker CM, Moore CG, Pape WJ, Eisen L. Seasonal patterns for entomological measures of risk for exposure to Culex vectors and West Nile virus in relation to human disease cases in northeastern Colorado. J Med Entomol 2009;46(6):1519–31. 10.1603/033.046.0641 19960707 PMC2802831

[52] Kilpatrick AM, Pape WJ. Predicting human West Nile virus infections with mosquito surveillance data. Am J Epidemiol 2013;178(5):829–35. 10.1093/aje/kwt046 23825164 PMC3755645

[53] Thomas-Bachli AL, Pearl DL, Berke O, Parmley EJ, Barker IK. A comparison of West Nile virus surveillance using survival analyses of dead corvid and mosquito pool data in Ontario, 2002-2008. Prev Vet Med 2015;122(3):363–70. 10.1016/j.prevetmed.2015.10.007 26520177

[54] Karki S, Westcott NE, Muturi EJ, Brown WM, Ruiz MO. Assessing human risk of illness with West Nile virus mosquito surveillance data to improve public health preparedness. Zoonoses Public Health 2018;65(1):177–84. 10.1111/zph.12386 28816022

